# Structural insights into cyanobacterial RuBisCO assembly coordinated by two chaperones Raf1 and RbcX

**DOI:** 10.1038/s41421-022-00436-9

**Published:** 2022-09-20

**Authors:** Qiong Li, Yong-Liang Jiang, Ling-Yun Xia, Yuxing Chen, Cong-Zhao Zhou

**Affiliations:** School of Life Sciences, University of Science and Technology of China, Hefei, Anhui China

**Keywords:** Cryoelectron microscopy, Chaperones

## Abstract

RuBisCO is the most abundant enzyme in nature, catalyzing the fixation of CO_2_ in photosynthesis. Its common form consists of eight RbcL and eight RbcS subunits, the assembly of which requires a series of chaperones that include RbcX and RuBisCO accumulation factor 1 (Raf1). To understand how these RuBisCO-specific chaperones function during cyanobacterial RbcL_8_RbcS_8_ (L_8_S_8_) holoenzyme formation, we solved a 3.3-Å cryo-electron microscopy structure of a 32-subunit RbcL_8_Raf1_8_RbcX_16_ (L_8_F_8_X_16_) assembly intermediate from *Anabaena* sp. PCC 7120. Comparison to the previously resolved L_8_F_8_ and L_8_X_16_ structures together with biochemical assays revealed that the L_8_F_8_X_16_ complex forms a rather dynamic structural intermediate, favoring RbcS displacement of Raf1 and RbcX. In vitro assays further demonstrated that both Raf1 and RbcX function to regulate RuBisCO condensate formation by restricting CcmM35 binding to the stably assembled L_8_S_8_ holoenzymes. Combined with previous findings, we propose a model on how Raf1 and RbcX work in concert to facilitate, and regulate, cyanobacterial RuBisCO assembly as well as disassembly of RuBisCO condensates.

## Introduction

Life on earth depends on the photosynthesis pathway to convert atmospheric CO_2_ into organic carbon. This process is initiated by the globally most abundant enzyme ribulose-1,5-bisphosphate carboxylase/oxygenase (RuBisCO), whose total mass in nature is ~0.7 Gt^1,2^. As the most common form, the form I RuBisCO in plants, eukaryotic algae, and cyanobacteria is a ~530 kDa complex consisting of eight large (RbcL, ~53 kDa) and eight small (RbcS, ~15 kDa) subunits^3^. Eight RbcL subunits are assembled into a tetramer of catalytic antiparallel dimers, which are capped by four RbcS subunits at the top and bottom, respectively, forming a functional holoenzyme RbcL_8_RbcS_8_ (L_8_S_8_). Remarkably, RuBisCO is a rather inefficient and error-prone enzyme due to its slow catalytic rate (~3–12 s^−1^) and limited specificity towards CO_2_ versus O_2_^4,5^.

Generally, RuBisCO biogenesis is a complicated process that requires a series of molecular chaperones^6–8^. In cyanobacteria, the nascent RbcL subunits are initially folded by the chaperonin GroEL-GroES^9^, followed by assembly of the octameric core RbcL_8_, mainly assisted by individual chaperones such as RuBisCO accumulation factor Raf1^7,10^ and RbcX^11–13^. Afterward, docking of RbcS subunits displaces Raf1^14^ and/or RbcX^13^ to enable the formation of L_8_S_8_ holoenzyme. The previously solved RbcL_8_RbcX_16_ (L_8_X_16_) structure showed that RbcX is a homodimer of mostly α-helical structure with a central cleft binding to the C-terminal conserved motif of RbcL^13^. Our previously reported structures of Raf1 and its complex with RbcL (RbcL_8_Raf1_8_, termed L_8_F_8_ for short) demonstrated that Raf1 is also a homodimer, each subunit of which consists of an N-terminal α-helical domain (Raf1α) and a C-terminal β-sheet dimerization domain (Raf1β) separated by a flexible linker^10^. Upon binding to RbcL, the Raf1α and Raf1β domains of Raf1 undergo rigid body rotations to embrace an RbcL dimer, forming the complex L_8_F_8_, in which Raf1β are arranged around the equator of each RbcL dimer, whereas the two Raf1α domains contact the top and bottom edges of the RbcL dimer^10^. Given the co-existence of Raf1 and RbcX in most cyanobacteria and plants^15^, the two chaperones might function in concert on RuBisCO assembly. However, how these two chaperones interplay on RuBisCO biogenesis remains elusive.

Here we solved the 3.3-Å cryo-electron microscopy (cryo-EM) structure of a ternary complex composed of RbcL, Raf1, and RbcX, representing a cyanobacterial RuBisCO assembly intermediate. Structural and biochemical analyses elucidated the mechanism underlying the concerted action of Raf1 and RbcX on the assembly of RuBisCO holoenzyme and disassembly of RuBisCO condensates. All these findings provide new insights into cyanobacterial RuBisCO assembly and potential avenues for its engineering in heterologous systems toward improving plant photosynthesis and growth^16,17^.

## Results

### Structure of the RuBisCO assembly intermediate RbcL_8_Raf1_8_RbcX_16_

**Fig. 1:**
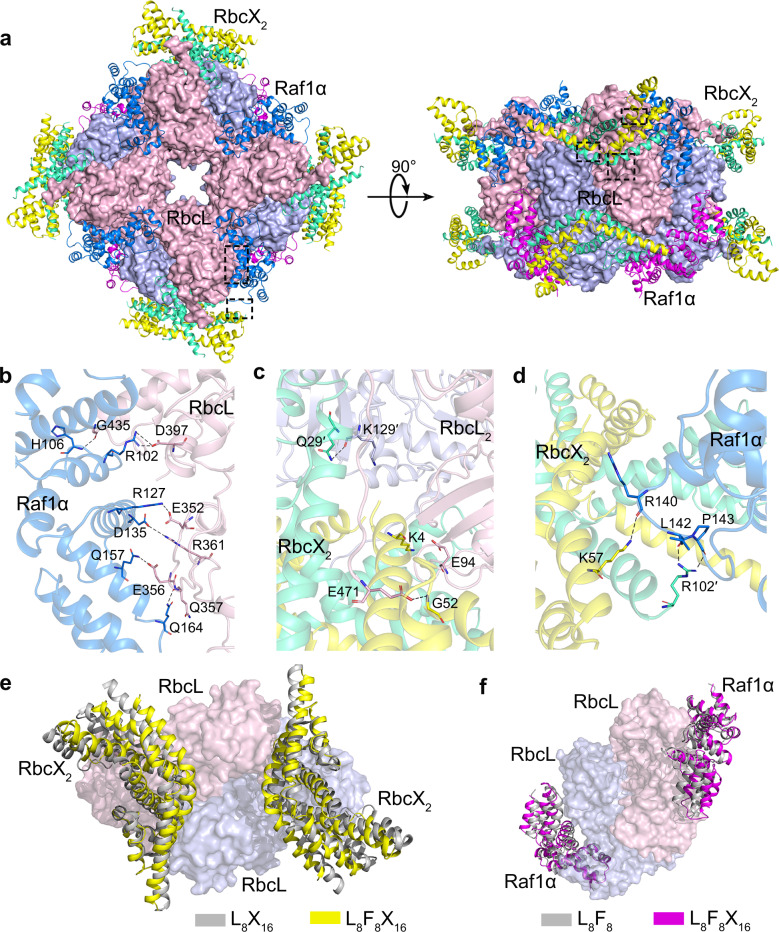
Cryo-EM structure of L_8_F_8_X_16_. **a** The overall structure of L_8_F_8_X_16_ is shown in two orientations rotated by 90°. The RbcL octameric core is shown as the surface, with the two subunits of each dimer colored in pink and blue, respectively. The four Raf1α dimers and the eight RbcX dimers are shown as cartoons. The two subunits of each Raf1α dimer are respectively colored in marine and magenta, whereas those of each RbcX dimer are respectively colored in yellow and green. The interfaces between RbcL–Raf1α, RbcL–RbcX, and Raf1α–RbcX are indicated by dashed boxes. **b**–**d** The three interfaces of RbcL–Raf1α (**b**), RbcL–RbcX (**c**), and RbcX–Raf1α (**d**) in L_8_F_8_X_16_. The color scheme of each subunit is the same as that in the overall structure of L_8_F_8_X_16_. RbcL, Raf1α, and RbcX are shown as semi-transparent cartoons. The interacting residues are shown as sticks and labeled, with hydrogen bonds indicated as dashed lines. **e** Structural comparison of two RbcX dimers in the structures of L_8_F_8_X_16_ and L_8_X_16_ (PDB, 3RG6). **f** Structural comparison of Raf1α domains in the structures of L_8_F_8_X_16_ and L_8_F_8_ (PDB, 6KKM).

We co-expressed RbcL with the chaperones Raf1 and RbcX from *Anabaena* sp. PCC 7120, in the presence of the chaperonin GroEL-GroES, which facilitates the formation of a 32-subunit RbcL–Raf1–RbcX ternary complex of ~1 MDa in size (Supplementary Fig. S1). Subsequently, we purified this complex and solved its cryo-EM structure at 3.3 Å resolution (Fig. 1a; Supplementary Fig. S2a–c), representing an intermediate of RbcL_8_ core engaged by 24 molecules of chaperones. The overall density map, especially RbcL_8_ core at the center, is of high quality, whereas the surrounding regions corresponding to RbcX are relatively dispersed (Supplementary Fig. S2d). Nevertheless, thanks to the known structures of RbcL, Raf1, and RbcX^10,13^, we successfully fitted all protein components into the map and finally obtained the complex structure RbcL_8_Raf1_8_RbcX_16_, termed L_8_F_8_X_16_ for short (Fig. 1a). This structure demonstrates the interaction patterns of a RuBisCO intermediate bound by multiple chaperones.

In the L_8_F_8_X_16_ structure, eight RbcL subunits form a core of four antiparallel dimers (Fig. 1a), which resembles the RbcL_8_ octameric core seen previously in RuBisCO-chaperone intermediary structures^10,13,18^. Similar to the L_8_F_8_ structure^10^, each Raf1α domain of Raf1 is docked onto the interface cleft between two neighboring RbcL dimers, embracing the C-terminal TIM-barrel domain of an RbcL subunit (Fig. 1b; Supplementary Table S1). Additionally, Raf1α also makes contact with the N-terminal domain of the adjacent RbcL dimer (Fig. 1b; Supplementary Table S1). However, the Raf1β domain of Raf1, which binds to the equator of RbcL_8_ in the structure of L_8_F_8_^10^, could not be modeled in the final map of L_8_F_8_X_16_ due to the untraceable density. Moreover, similar to the chimeric structure of L_8_X_16_ composed of *Synechococcus* sp. PCC 6301 RbcL and *Anabaena* sp. CA RbcX^13^, two RbcX dimers are docked to one RbcL dimer via interacting with the C-terminal peptide of one RbcL subunit and the N-terminal domain of the other subunit (Fig. 1c; Supplementary Table S1). Of note, Raf1α also interacts with one subunit of RbcX dimer (RbcX_2_) via several hydrogen bonds, including Raf1^R140^–RbcX^K57^, Raf1^L142^–RbcX^R102^, Raf1^P143^–RbcX^R102^ bonds, to further stabilize the complex (Fig. 1d; Supplementary Table S1). In sum, the ternary complex of L_8_F_8_X_16_ has three different interfaces, which possess a buried interface area of ~740, 1000, and 400 Å^2^ for RbcL–Raf1α, RbcL–RbcX_2_, and Raf1–RbcX_2_, respectively. Sequence analyses showed that the interface residues within RbcL–Raf1 and RbcL–RbcX are highly conserved, whereas the residues at the Raf1–RbcX interface are relatively variable among Raf1 and RbcX homologs (Supplementary Fig. S3). It indicates that Raf1 and RbcX do not possess specific interactions with each other and are respectively recruited by RbcL.

Compared to L_8_F_8_ and L_8_X_16_, the L_8_F_8_X_16_ structure exhibits significant conformational variations, beyond similar architectures and binding patterns. In contrast to L_8_X_16_, each RbcX_2_ moves ~2.6 Å towards the equator of RbcL dimer in L_8_F_8_X_16_, forming an interface area of ~1000 Å^2^ between RbcL and RbcX_2_ in L_8_F_8_X_16_, compared to that of ~1400 Å^2^ in L_8_X_16_ (Fig. 1e). The structural comparison showed that Raf1β in L_8_F_8_ and RbcX_2_ in L_8_F_8_X_16_ share a partially overlapped binding region on RbcL (Supplementary Fig. S4). Thus Raf1β is expelled from the equator of RbcL_8_ upon RbcX binding, which might be flexibly tethered nearby the RbcL_8_ core, due to the 23-residue linker between Raf1α and Raf1β. Moreover, Raf1α in L_8_F_8_X_16_ moves ~3.0 Å away from the RbcL_8_ core compared to that in L_8_F_8_ (Fig. 1f), resulting in a dramatic decrease of RbcL–Raf1α interface from ~1000 Å^2^ in L_8_F_8_ to ~740 Å^2^ in L_8_F_8_X_16_. Another notable difference is the 8-residue C-terminal tail of Raf1 (C-tail), which inserts deeply into the active-site pocket of RbcL in L_8_F_8_^10^, and is untraceable in L_8_F_8_X_16_. Superposition of L_8_F_8_ onto L_8_F_8_X_16_ revealed that Raf1 C-tail and one subunit of RbcX_2_ have a large steric hindrance (Supplementary Fig. S4). Notably, similar to that in L_8_X_16_, the conserved C-terminal peptide of RbcL is embedded in a narrow hydrophobic groove of RbcX_2_ in L_8_F_8_X_16_, different from that interacting with Raf1α in L_8_F_8_ (Supplementary Fig. S5). Moreover, the so-called “60s loop” of RbcL (residues 64–83) that forms a part of the catalytic pocket, is missing in both structures of L_8_X_16_ and L_8_F_8_X_16_, whereas it is stabilized by Raf1α in L_8_F_8_.

Generally, the L_8_F_8_X_16_ structure shows much looser contacts of Raf1 or RbcX with RbcL, indicating that Raf1 and RbcX partly antagonize each other upon binding to RbcL_8_. As a consequence, L_8_F_8_X_16_ adopts a rather dynamic structure with a more relaxed RbcL_8_ core that comprises a 25 Å central pore in diameter, which is ~5 Å larger than those in either L_8_F_8_, L_8_X_16_ or L_8_S_8_ structures (Supplementary Fig. S6).

### Concerted action of Raf1 and RbcX on RuBisCO assembly

**Fig. 2:**
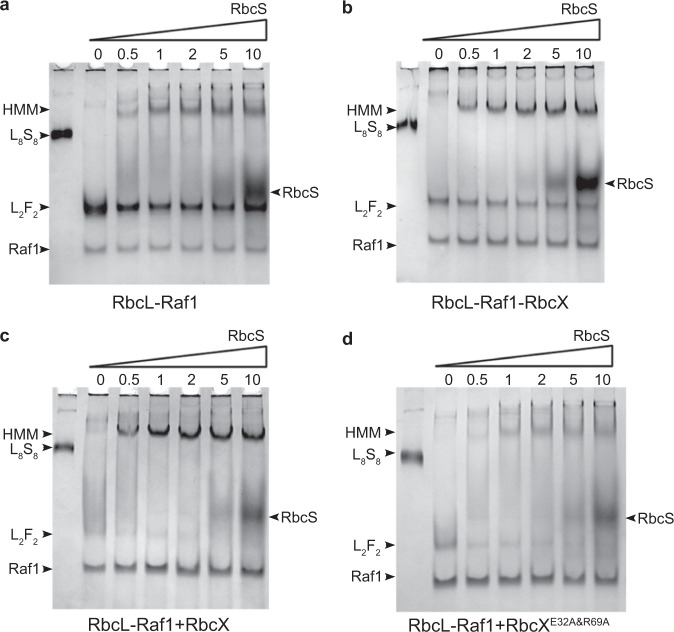
Native-PAGE analyses of RuBisCO assembly. **a**–**d** RbcS proteins at increasing concentrations were added to the solution of RbcL–Raf1 (**a**), RbcL–Raf1–RbcX (**b**), or RbcL–Raf1 pre-incubated with RbcX (**c**) or RbcX^E32A&R69A^ (**d**). The concentrations of RbcS are 0, 2, 4, 8, 20, and 40 μM, with the molar ratio of 0, 0.5, 1, 2, 5, and 10 fold RbcS (as shown at the top) to RbcL. The concentrations of RbcX and RbcX^E32A&R69A^ are 8 μM, which is 2-fold that of RbcL. The assembly intermediates are indicated by arrows on the left of the native-PAGE, in which HMM represents the complexes of high-molecular-mass intermediates. The *Anabaena* sp. PCC 7120 L_8_S_8_ holoenzyme was used as the positive control in lane 1 of each panel.

To test if this rather relaxed RbcL_8_ core is more favorable for RbcS recruitment, we performed in vitro RuBisCO assembly assays. Titration of RbcS at various ratios to the L_8_F_8_ complexes gradually triggered the formation of higher-molecular-mass (HMM) intermediates, corresponding to the ternary complex RbcL–Raf1–RbcS (Fig. 2a; Supplementary Fig. S7a). However, almost no RuBisCO holoenzyme could be detected, even in the presence of 10-fold RbcS in molarity to that of RbcL (Fig. 2a), indicating that excess RbcS could hardly displace Raf1 from L_8_F_8_, most likely due to the tight interaction between RbcL and Raf1^10^. By contrast, the addition of RbcS at increasing ratios into L_8_F_8_X_16_ solution gradually triggers the formation of HMM intermediates that contain RbcL, RbcS, Raf1, and RbcX (Fig. 2b; Supplementary Fig. S7b). Upon the addition of RbcS up to 10-fold in molarity to that of RbcL, these HMM intermediates remain heterogeneous and finally reach a migration rate close to that of L_8_S_8_ holoenzyme (Fig. 2b), accompanied by the decrease of Raf1 in the intermediates (Supplementary Fig. S7b). Moreover, titration of RbcS at various ratios to the L_8_F_8_ solution pre-incubated with 16-fold RbcX in molarity also yielded a similar profile of HMM intermediates formation (Fig. 2c; Supplementary Fig. S7c). In contrast, pre-incubation of RbcL–Raf1 complexes with RbcX^E32A&R69A^ mutant, in which the two RbcX residues directly interacting with RbcL^13^ were mutated, no longer promoted the formation of HMM intermediates that possess less Raf1 (Fig. 2d; Supplementary Fig. S7d), but led to the formation of HMM intermediates that behave quite similarly to that of RbcL–Raf1 complexes titrated with RbcS (Fig. 2a; Supplementary Fig. S7a).

Furthermore, we applied the RuBisCO carboxylase activity assays to further evaluate whether the functional RuBisCO active sites are formed in these HMM intermediates. As expected, the RbcL–Raf1 complexes were almost inactive without RbcS (Supplementary Fig. S8). Addition of RbcS at increasing concentrations to RbcL–Raf1 complexes gradually augmented the carboxylase activities (Supplementary Fig. S8), which indicates that displacement of Raf1 by RbcS enables the formation of functional RuBisCO active sites. However, even 10-fold molarity of RbcS could not completely displace Raf1 from RbcL, as shown by a much lower activity compared to that of *Synechococcus elongatus* PCC 7942 RuBisCO holoenzyme (Supplementary Fig. S8). Moreover, compared to RbcL–Raf1 complexes, pre-incubation with RbcX_2_ at equal molarity in vitro led to a drastic decrease in the carboxylase activity, in the presence of RbcS at the same molarity (Supplementary Fig. S8). Apparently, the addition of extra RbcX to RbcL–Raf1 complexes facilitates the release of Raf1 from RbcL, and leads to the formation of highly dynamic HMM complexes composed of a series of RuBisCO assembly intermediates, including RbcL–RbcX and RbcL–Raf1–RbcX complexes. In fact, under the physiological conditions, the relative abundance of Raf1 and RbcX is only ~0.6% or less than that of RbcL^19^; thus, the higher excess RbcS in cyanobacterial cells could easily displace Raf1 and/or RbcX from RbcL. Taken together, our results suggest that RbcX acts in concert with Raf1 to maintain the homeostasis of RuBisCO assembly, which is a rather dynamic and reversible process composed of various assembly intermediates.

Notably, compared to the dynamic RbcL–Raf1 complexes that have a larger fraction of L_2_F_2_ (Fig. 2a), the bands corresponding to L_2_F_2_ sharply decreased in the samples of RbcL–Raf1–RbcX complexes (Fig. 2b), and were almost diminished in the samples of RbcL–Raf1 complexes pre-incubated with RbcX (Fig. 2c). Upon the increase of RbcX in the RbcL–Raf1 complexes, followed by the addition of RbcS (1:1 molarity to RbcL), it yielded more HMM intermediates and fewer L_2_F_2_ complexes (Supplementary Fig. S9). Once the equal molarity of RbcX_2_ was added to the solution, almost all RbcL proteins become the HMM intermediates, with most Raf1 released and L_2_F_2_ undetectable (Supplementary Fig. S9). These results suggested that RbcX could also facilitate the shift of equilibrium from RbcL dimer to RbcL octamer, besides promoting the release of Raf1 from RbcL.

### RbcX can efficiently solubilize CcmM35-mediated RuBisCO condensates

**Fig. 3:**
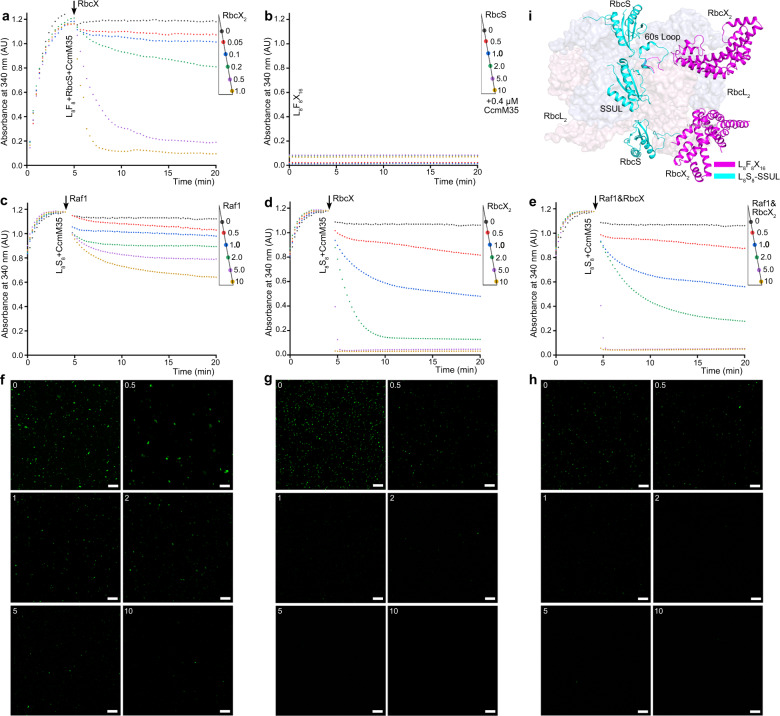
Effects of Raf1 and RbcX on solubilizing the CcmM35-mediated condensates of RuBisCO. **a** Plots of solubilizing *Anabaena* sp. PCC 7120 RuBisCO condensates upon addition of RbcX. The RuBisCO condensates were formed by mixing 0.5 µM L_8_F_8_, 4 µM RbcS, and 4 µM CcmM35 for 5 min. RbcX_2_ at various concentrations (0, 0.2, 0.4, 0.8, 2 and 4 μM), corresponding to a molar ratio of 0, 0.05, 0.1, 0.2, 0.5 and 1 fold RbcX_2_ to RbcL, respectively, was then added to the turbid solution. **b** Plots of condensate formation from *Anabaena* sp. PCC 7120 L_8_F_8_X_16_ was incubated with RbcS for 30 min, and then 4 µM of CcmM35. 0.5 µM L_8_F_8_X_16_ was incubated with RbcS at various concentrations (0, 2, 4, 8, 20 and 40 µM), corresponding to a molar ratio of 0, 0.5, 1, 2, 5 and 10 fold RbcS to RbcL, respectively. **c**–**e** Plots of *S. elongatus* PCC 7942 RuBisCO condensate solubilization upon adding Raf1 (**c**), RbcX (**d**) and Raf1&RbcX (**e**), respectively. The RuBisCO condensates were formed by mixing 0.25 µM L_8_S_8_ and 2 µM CcmM35 for 4 min. Raf1, RbcX_2_ and Raf1&RbcX_2_ at various concentrations (0, 1, 2, 4, 10 and 20 μM), corresponding to a molar ratio of 0, 0.5, 1, 2, 5, and 10 fold Raf1 or RbcX_2_ to RbcL, respectively, were then added to the turbid solution. The turbidity was monitored at 340 nm over time. **f**–**h** Confocal microscopy images of the final condensates corresponding to **c**–**e**. Scale bars, 20 μm. **i** Structural superposition of L_8_F_8_X_16_ against L_8_S_8_–SSUL complex (PDB, 6HBC). The RbcL dimer is shown as surface and colored in pink/blue. The SSUL domain and 60s loop of RbcL from L_8_S_8_–SSUL complex are indicated by cyan cartoon, whereas the Raf1α domains and 60s loops of RbcL from L_8_F_8_X_16_ are shown as a magenta cartoon.

In cyanobacteria, RuBisCO holoenzymes further form condensates that are cross-linked by the scaffold protein CcmM35, the truncated form of CcmM that harbors three RuBisCO small-subunit-like (SSUL) modules^20,21^. We previously found that Raf1 acts as a solubilizer that antagonizes CcmM35-mediated RuBisCO condensates formation in vitro^10^. When incubating *Anabaena* sp. PCC 7120 L_8_F_8_ complex with 8-fold RbcS in molarity, followed by the addition of 8-fold CcmM35 in solution, we prepared the RuBisCO condensates, as shown by that the turbidity finally reached the maximum absorbance (Fig. 3a). Afterward, the addition of RbcX triggers the decrease in turbidity over time (Fig. 3a), indicating the gradual disassembly of the condensates. The more RbcX that was added, the faster the condensates were solubilized. Once adding RbcX_2_ in equal molarity to that of RbcL, the solution became clear in ~10 min (Fig. 3a), indicating that RuBisCO condensates were almost completely solubilized. Moreover, the L_8_F_8_X_16_ complexes could no longer form RuBisCO condensates even in the presence of excess RbcS up to 10-fold in molarity to that of RbcL (Fig. 3b).

To further compare the effect of Raf1 and RbcX on solubilizing RuBisCO condensates, we prepared RuBisCO condensates by using *S. elongatus* PCC 7942 L_8_S_8_, due to that the insufficient amount of *Anabaena* sp. PCC 7120 L_8_S_8_ is required for multiple rounds of in vitro assays. As a previously reported solubilizer^10^, Raf1 could trigger the disassembly of the RuBisCO condensates, as shown by the gradual decrease in turbidity over time (Fig. 3c); however, it is of relatively lower efficiency, as even 10-fold Raf1 could only partially solubilize the condensates. In contrast, RbcX showed a much higher efficiency compared to Raf1, as the RuBisCO condensates were almost completely solubilized in ~5 min upon addition of 5-fold RbcX_2_ (Fig. 3d). Notably, simultaneous addition of both Raf1 and RbcX_2_ (at equal molarity) showed a profile of quick decrease of turbidity over time (Fig. 3e), similar to that upon addition of RbcX_2_ alone.

Moreover, we applied confocal fluorescence spectroscopy assays to observe the disassembly of RuBisCO condensates upon the addition of Raf1 and/or RbcX. Similar to the turbidity assays, a small fraction of RuBisCO condensates still existed even 10-fold molarity of Raf1 was added to the turbid solution, further confirming that Raf1 is a solubilizer with low efficiency (Fig. 3f). In contrast, the RuBisCO condensates were almost completely solubilized upon the addition of RbcX_2_ (Fig. 3g) or Raf1/RbcX_2_ at 5-fold molarity (Fig. 3h). These results suggested that both Raf1 and RbcX could solubilize the RuBisCO condensates in vitro under the tested conditions, and RbcX possesses a much higher efficiency.

It was previously reported that Raf1 antagonizes the RuBisCO condensation via competitively binding to the SSUL-binding site on RbcL^10^. Despite RbcX possessing a binding site on RbcL different from that of SSUL, the 60s loop of RbcL is disordered upon binding to RbcX, as shown in the structure of either L_8_X_16_^13^ or L_8_F_8_X_16_ (Fig. 3i). It was known that the 60s loop of RbcL directly interacts with SSUL in the structure of RuBisCO–SSUL^21^, and is necessary for the recruitment of RbcS to form RuBisCO holoenzyme^22^. Thus upon binding to RbcX, RbcL might adopt an altered conformation that is incapable of binding to RbcS and CcmM35-SSUL, which are prerequisites for the assembly of RuBisCO holoenzymes and succeeding condensation.

## Discussion

**Fig. 4:**
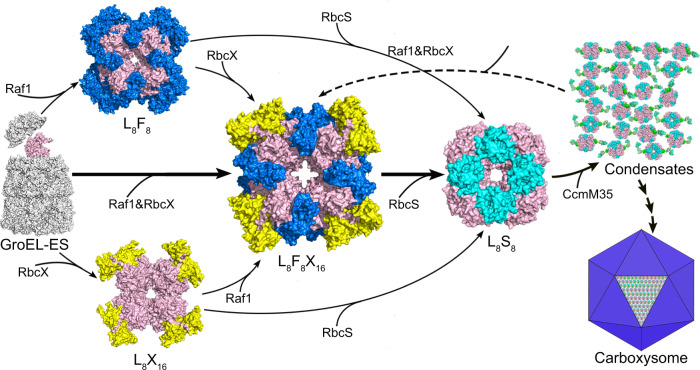
An updated model of cyanobacterial RuBisCO assembly assisted by Raf1 and RbcX. The previously identified paths of individual Raf1- or RbcX-assisted RuBisCO assembly are also included in the model. The proteins GroEL-GroES, RbcL, RbcS, Raf1, and RbcX are shown as surfaces and are colored in gray, pink, cyan, marine, and yellow, respectively. A putative step of condensates disassembly is indicated by the dashed line.

Combined with previous findings of Raf1^7,10,15,23^ and RbcX^11–13,24^, the present ternary structure of L_8_F_8_X_16_ intermediate together with biochemical assays provide fine molecular insights into the coordinated action of Raf1 and RbcX on RuBisCO holoenzyme assembly and CcmM35-mediated condensates disassembly (Fig. 4). Assembly of RbcL_8_ core could be assisted by the individual chaperone Raf1 or RbcX (Fig. 4, the upper and lower paths). However, in most cases, Raf1 and RbcX could simultaneously bind to RbcL, forming a dynamic L_8_F_8_X_16_ intermediate (Fig. 4, the central path), which favors RbcS displacement of the two chaperones. Notably, as an early-stage assembly chaperone^7^, Raf1 is a major contributor to forming the RbcL dimers in the form of L_2_F_2_, in addition to L_8_F_8_ (Fig. 2a). Given a much larger interface of RbcL–Raf1, compared to a smaller interface of RbcL–RbcX (Fig. 1b, c), it is possible that Raf1 functions as a major contributor in RuBisCO assembly since its deletion in cyanobacteria and plants precludes RuBisCO biogenesis^15^, whereas RuBisCO production in cyanobacteria is unaffected by RbcX deletion^25^. Moreover, plant RuBisCO biogenesis in *Escherichia coli* remains feasible upon RbcX omission, but fully reliant on Raf1 production^26^. Our biochemical assays showed that similar to the C-terminal tail of Raf1^10^, RbcX also facilitates the formation of RbcL octamers from dimers in the presence of Raf1 (Fig. 2c; Supplementary Fig. S9). Thus RbcX assists and acts in concert with Raf1 to form a dynamic L_8_F_8_X_16_ intermediate, eventually favoring RbcS recruitment, indicating that RbcX is more likely an assistant chaperone that functions at the relatively late stage.

The ordered RuBisCO holoenzyme structure with a tightly packed RbcL_8_ core is a prerequisite for the proper RuBisCO lattice formation and succeeding recruitment of shell proteins of carboxysome^27–29^. Beyond functioning as a chaperone, RbcX is a more efficient solubilizer to dissolve the RuBisCO condensates in vitro under tested conditions, in contrast to Raf1 (Fig. 3). It implies that RbcX might be a key regulator that controls RuBisCO condensation, a process succeeding the maturation of RuBisCO holoenzymes in cyanobacteria. In fact, a previous report suggested that RbcX appears as one component of carboxysome and co-localizes with RuBisCO which mediates carboxysome biogenesis in vivo^24^.

Introducing cyanobacterial carboxysomes into plant chloroplasts has become a promising strategy for genetic engineering to improve photosynthetic performance^30–32^. However, reconstituting entire functional β-carboxysomes in heterologous hosts is still a big challenge, partly due to the sophisticated mechanisms of RuBisCO assembly and carboxysome biogenesis. Notably, the plant RuBisCO assembly requires a chloroplast-specific chaperone BSD2^33^, which was proposed to be an end-stage assembly factor of RuBisCO^26^ and acts as a negative regulator of RbcL transcription^34,35^. It was proposed that in the chloroplast, BSD2 may have diminished the role of RbcX in RuBisCO assembly, owing to a partly overlapped binding site on RbcL^26^. In addition, cyanobacterial Raf1 may serve dual functions of both plant Raf1 and BSD2, where the two conserved C-terminal acidic residues of either BSD2 or cyanobacterial Raf1 insert into the RbcL catalytic pocket, contributing to RbcL octamer assembly^10^. Given a more complicated biogenesis pathway of plant L_8_S_8_ holoenzyme, further studies are needed to elucidate the fine mechanism by which BSD2 might provide an adaptive advantage during plant RuBisCO biogenesis and/or repair.

Despite that cyanobacteria and plants share a generally similar mechanism of RuBisCO assembly, the roles of individual chaperones differ a lot. To guarantee the proper assembly of carboxysome in the chloroplast, it is feasible to introduce cyanobacterial chaperones Raf1 and RbcX into plants, especially when cyanobacterial RuBisCO is incorporated. Notably, introducing *S. elongatus* PCC 7942 RuBisCO, together with RbcX and CcmM35 into tobacco chloroplast supported the autotrophic photosynthesis^16^, which provided an initial successful trial of replacing plant RuBisCO. However, for proper assembly and functionality of carboxysome in C3 plants, more investigations are needed to comprehend the coordinated action of cyanobacterial and chloroplast-specific chaperones on RuBisCO assembly and carboxysome biogenesis in plants.

In sum, our present findings, together with previous reports, enable us to better understand the molecular insights into RuBisCO assembly and carboxysome biogenesis in different cyanobacterial strains^10,15,24,25,36,37^. The chaperones Raf1 and RbcX act in concert to regulate multiple stages of cyanobacterial RuBisCO assembly and condensation. Formation of a dynamic ternary complex L_8_F_8_X_16_, via simultaneous binding of Raf1 and RbcX to RbcL, facilitates the release of RbcL_8_ core from the chaperones. Moreover, beyond functioning as a late-stage assembly factor to promote the formation of RbcL_8_ core, RbcX is a major contributor to regulating the dynamic balance between RuBisCO holoenzymes and condensates. These findings provide an advanced understanding of the fine functions of cyanobacterial chaperones Raf1 and RbcX in RuBisCO assembly and carboxysome formation, which will guide the design and engineering of a more efficient RuBisCO and/or carboxysome in plants for enhanced carbon fixation and agricultural productivity.

## Materials and methods

### Cloning and plasmids

The coding regions of RbcL, Raf1, RbcX, RbcS, GroEL-GroES, and CcmM35 were amplified from the genomic DNA of cyanobacteria *Anabaena sp*. PCC 7120 or *S. elongatus* PCC 7942, and were cloned into the pET19 and/or pCDFDuet vectors using the homologous recombination methods. The details of plasmids and protein sequences used in this study are listed in Supplementary Table S2.

### Protein expression and purification

The ternary complex of *Anabaena* sp. PCC 7120 RbcL–Raf1–RbcX was obtained by co-expressing the plasmids of pCDFduet-GroEL-GroES-RbcX-Raf1 and pET19-His-RbcL in *E. coli* BL21 (DE3) strain. The transformed cells were cultured at 37 °C in 6 L Luria-Bertani medium (10 g NaCl, 10 g Bacto Tryptone, and 5 g yeast extract/L) containing ampicillin of 50 μg/mL and spectinomycin of 100 μg/mL to an A_600 nm_ of 0.8, and then induced with 0.2 mM isopropyl-β-D-thiogalactoside for a further 20 h at 16 °C. The cells were harvested by 5 min of centrifugation at 8000× *g*, resuspended in 40 mL lysis buffer (50 mM Tris-HCl, pH 8.0, 20 mM NaCl, 5 mM MgCl_2_), and disrupted by the Ultrasonic Cell Disruptor (SONICS). After centrifugation at 12,000× *g* for 30 min, the supernatant was loaded onto a Ni-NTA column (Qiagen) pre-equilibrated with the binding buffer (50 mM Tris-HCl, pH 8.0, 20 mM NaCl, 5 mM MgCl_2_). The target protein was eluted with the binding buffer containing 500 mM imidazole, and further purified by gel filtration (Superdex 200 Increase 10/300, GE Healthcare) in the binding buffer. The peak fractions containing the complex of RbcL–Raf1–RbcX were collected by monitoring the absorbance at 280 nm, and concentrated to 2 mg/mL for cryo-EM analysis or 10 mg/mL for biochemical assays by 100 kDa cut-off concentrators.

The *Anabaena* sp. PCC 7120 RbcL–Raf1 complex was obtained by co-transforming the plasmids of pET19-His-RbcL–Raf1 and pCDFduet-GroEL-GroES into *E. coli* (DE3) strain. The cells were cultured, overexpressed, and purified following a previously described protocol^10^. The target proteins were flash-frozen in liquid nitrogen and stored at −80 °C for further use.

The *Anabaena* sp. PCC 7120 RuBisCO holoenzyme was obtained by co-purifying the Flag-RbcS and RbcL–Raf1–RbcX complex, in which the Raf1 protein is from *S. elongatus* PCC 7942. The Flag-RbcS and RbcL–Raf1–RbcX complex were overexpressed in *E. coli* BL21 (DE3) cells with transformed plasmids of pET19-Flag-RbcS and pCDFduet-GroEL-GroES-RbcX-Raf1/pET19-His-RbcL, respectively. The harvested cells were mixed and disrupted by the Ultrasonic Cell Disruptor (SONICS). The following purification procedures were the same as that described for *Anabaena* sp. PCC 7120 RbcL–Raf1–RbcX complex. Notably, the purified *Anabaena* sp. PCC 7120 RuBisCO holoenzyme is only of little amount, which was only applicable in the native-PAGE analysis as a control marker.

The recombinant *Anabaena* sp. PCC 7120 RbcX, RbcX^E32A&R69A^, RbcS, and CcmM35 proteins were overexpressed in *E. coli* BL21 (DE3) strain using the plasmids of pET19-His-RbcX, pET19-His-RbcX^E32A&R69A^, pET19-His-RbcS, and pET19-His-CcmM35, respectively. They were purified and stored as described previously^10^. The recombinant *Anabaena* sp. PCC 7120 Flag-RbcS protein was expressed in *E. coli* BL21 (DE3) strain by transforming plasmid pET19-Flag-RbcS, and purified via affinity chromatography with anti-FLAG M2 gel (Sigma) and size-exclusion chromatography with Superdex 75 Increase 10/300 (GE Healthcare). These proteins were concentrated to 10 mg/mL for biochemical assays by centrifugation with 10 kDa cut-off concentrators.

The recombinant proteins of *S. elongatus* PCC 7942 RuBisCO holoenzyme, eGFP-RuBisCO, Raf1, RbcX, and CcmM35 were overexpressed, purified, and concentrated the same as those described in our previous report^10^.

The protein concentration was determined using a NanoDrop (Thermo Fisher Scientific), and the purity was assessed by SDS-PAGE.

### Cryo-EM sample preparation, data collection, and processing

The purified ternary complex of *Anabaena* sp. PCC 7120 RbcL–Raf1–RbcX was concentrated to ~2 mg/mL. An aliquot of 3.5 μL of the sample was applied to glow-discharged Quantifoil R1.2/1.3 300-mesh Cu Holey Carbon Grids. The grids were blotted for 4 s with a blot force of 2 and a wait time of 20 s, and then plunged into liquid ethane using a Vitrobot Mark IV (FEI) at 4 °C and 100% humidity. The cryo-EM data sets were collected by a 300 keV Titan Krios electron microscope (FEI) at the Center for Integrative Imaging, University of Science and Technology of China. Totally, 1780 micrograph stacks (32 frames, each 0.17 s, 9 e/Å^2^/s, total dose ~50 e/Å^2^) were recorded with a K2 Summit direct electron detector (Gatan) at the super-resolution mode in a nominal magnification of 22,500× with a defocus range from −1.0 to −2.0 μm. All stacks were motion-corrected and dose weighted using MotionCor2 (version 1.3.1)^38^, and binned 2-fold to yield a pixel size of 1.01 Å. The defocus values were estimated using CTFFIND4 (version 4.1)^39^.

After manual removal of bad images, a total of 308,714 particles were automatically picked from 1538 images using RELION (version 3.0)^40^. Then, these particles were boxed and binned 4-fold for 2D classification. 207,936 particles from all good classes were subjected to the 3D classification with C4 symmetry, during which particles were classified into 4 classes. 54,260 particles from one class that shows clear features of the ternary complex were re-extracted without binning for the 3D auto-refinement with D4 symmetry, yielding a density map with an overall resolution of 3.3 Å after post-processing.

Model building of L_8_F_8_X_16_ was performed using Chimera^41^ by manually fitting the L_8_F_8_ structure (PDB, 6KKM) into the map. The Raf1β domains were not modeled in the final structure due to the dispersed density. Moreover, the Raf1α domains were manually adjusted to best fit the map. Then, the structure of RbcX was manually built into the map using Chimera by fitting the *Anabaena* sp. CA RbcX structure (PDB, 2PEO) into the extra density of the map. The model was manually refined by COOT (version 0.8.9)^42^, followed by the iterative positional and B-factor refinement in real space using PHENIX (version 1.14)^43^. The final structure showed good geometry and was further evaluated using MolProbity^44^ (http://molprobity.biochem.duke.edu).

The flowchart of cryo-EM data processing is shown in Supplementary Fig. S10. The parameters of cryo-EM data collection, processing, structure determination, and refinement are listed in Supplementary Table S3.

### RuBisCO assembly assays

The purified L_8_F_8_, L_8_F_8_X_16_, RbcX, RbcX^E32A&R69A^, and Flag-RbcS were concentrated to 15, 15, 10, 10, and 3 mg/mL, respectively, for the RuBisCO assembly assays. All measurements were performed at 25 °C in the buffer containing 20 mM Tris-HCl, pH 8.0, 300 mM NaCl, 5 mM MgCl_2_, 5% glycerol. First, 0.5 µM L_8_F_8_ in the absence of RbcX_2_ or L_8_F_8_X_16_ alone was added to the solution containing RbcS at various concentrations (0, 0.5, 1, 2, 5, 10-fold to RbcL). Then, after incubation for about half an hour, 10 μL of each sample was mixed with the loading buffer and applied to native-PAGE analysis (6% Bis-Tris and boric acid). The protein compositions in the corresponding bands of the native-PAGE were further analyzed by SDS-PAGE. Moreover, 0.5 µM RbcL–Raf1 was pre-incubated with 8 µM RbcX or RbcX^E32A&R69A^ (2 fold to RbcL) for half an hour before being added to the solution containing RbcS at various concentrations, and then applied to native-PAGE analyses. In addition, 0.5 µM RbcL–Raf1 in the presence of RbcX_2_ at various concentrations (0, 0.05, 0.1, 0.2, 0.5, 1 fold to RbcL) was added to the solution containing 4 µM RbcS, and also applied to native-PAGE analyses. The purified *Anabaena* sp. PCC 7120 RuBisCO holoenzyme was applied to the native-PAGE as a positive control, and the purified RbcL–Raf1, RbcL–Raf1-RbcX, RbcX_His_ and Flag-RbcS proteins were also applied to the SDS-PAGE as positive controls.

### RuBisCO carboxylase activity assays

The HMM complexes analyzed by native-PAGE were also applied to the RuBisCO carboxylase activity assays. First, the RbcL–Raf1 solutions with or without RbcX_2_ at equal molarity were mixed with RbcS at increasing concentrations (0, 0.5, 1, 2, 5, 10-fold to RbcL). After incubation of the 100 µL reaction mixture for ~0.5 h at 25 °C, 10 μL of each sample was applied to the RuBisCO carboxylase activity assays. The amount of RbcL, corresponding to the number of active sites, is equal in all samples. The RuBisCO holoenzyme, which was used as a positive control, also contains the same amount of RbcL proteins. All the assays were tested at 25 °C for the RuBisCO carboxylase activity, using a commercial RuBisCO assay kit (BC0445, Solarbio Life Science Co., Beijing, China) according to the instruction of the manufacturer. Using a Beckman DU800 spectrophotometer, the RuBisCO carboxylase activity was measured at 340 nm in the unit of U/mg, which represents the oxidation of 1 nmol of NADH per min. The activity of each sample was repeated for three times.

### Turbidimetric assays

The turbidity of the solution was measured by monitoring the absorbance at 340 nm using a Beckman DU800 spectrophotometer. The purified *Anabaena* sp. PCC 7120 L_8_F_8_, L_8_F_8_X_16_, CcmM35, RbcX_2_ and RbcS proteins were concentrated to 15, 10, 10, 10 and 5 mg/mL, respectively, for the turbidimetric assays. All measurements were performed at 25 °C in the buffer containing 20 mM Tris-HCl, pH 8.0, 50 mM NaCl, 5 mM MgCl_2_. Two groups of experiments were designed to detect the effect of RbcX on RuBisCO condensate formation. First, the RuBisCO condensation was triggered by mixing 0.5 µM L_8_F_8_, 4 µM RbcS, and 4 µM CcmM35. After the condensates reached the maximum turbidity in ~5 min, RbcX_2_ at various concentrations (0, 0.2, 0.4, 0.8, 2, and 4 µM) was added to the solution. Second, 0.5 µM L_8_F_8_X_16_ was added in the solution containing RbcS at various concentrations (0, 2, 4, 8, 20 and 40 µM). After incubation for ~30 min, 4 µM CcmM35 was added to the solution. During the experiments, the absorbance at 340 nm was monitored along the time to indicate turbidity. The RbcL–Raf1 complexes without RbcS and CcmM35 proteins, which could not form turbidity, were used as a negative control.

The purified *S. elongatus* PCC 7942 RuBisCO holoenzyme, CcmM35, Raf1, and RbcX_2_ proteins were concentrated at 12, 10, 20, and 20 mg/mL, respectively, for the assays. All purified proteins were assessed by SDS-PAGE (Supplementary Fig. S11). All measurements were performed at 25 °C in the buffer containing 20 mM Tris-HCl, pH 8.0, 50 mM NaCl, 5 mM MgCl_2_. The RuBisCO condensation was triggered by mixing 0.25 µM RuBisCO and 2 µM CcmM35. After the condensates reached the maximum turbidity in ~4 min, Raf1, RbcX_2_, and Raf1&RbcX_2_ at various concentrations (0, 1, 2, 4, 10, and 20 µM) were added to the solution, and the turbidities were monitored at 340 nm over time. The graphs were plotted using the Origin Pro software.

### Laser-scanning confocal microscopy

Three groups of condensation experiments were performed in the same manner as described for the turbidimetric assays. However, the *S. elongatus* PCC 7942 RuBisCO was replaced with eGFP-RuBisCO, in which an eGFP tag was fused to the N-terminus of RbcL. After incubating for 20 min, the reaction mixtures were immediately imaged by a laser-scanning confocal microscope (ZEISS LSM710). The 20 μL samples were transferred to a glass-bottom cell culture dish and excited with a laser at 488 nm for green fluorescence imaging. Images were recorded by focusing on the bottom of the dish using Axio Observer Z1 microscope with a Plan-Apochromat 20×/0.8 M27 objective.

## Supplementary information

Supplementary Figures and Tables

## Data Availability

The cryo-EM structure of L_8_F_8_X_16_ has been deposited at PDB under the accession code of 7XSD. The cryo-EM density map of L_8_F_8_X_16_ has been deposited at the Electron Microscopy Data Bank (EMD-33524).
