# Supplementary material for: Structural insights into cyanobacterial RuBisCO assembly coordinated by two chaperones Raf1 and RbcX
Source: Cell Discov. 2022 Sep 20;8:93. doi: 10.1038/s41421-022-00436-9 (PMC9485235; doi:10.1038/s41421-022-00436-9)
Supplement: Supplementary file 1 — Supplementary Figures and Tables [file 41421_2022_436_MOESM1_ESM.pdf]

Supplementary Information

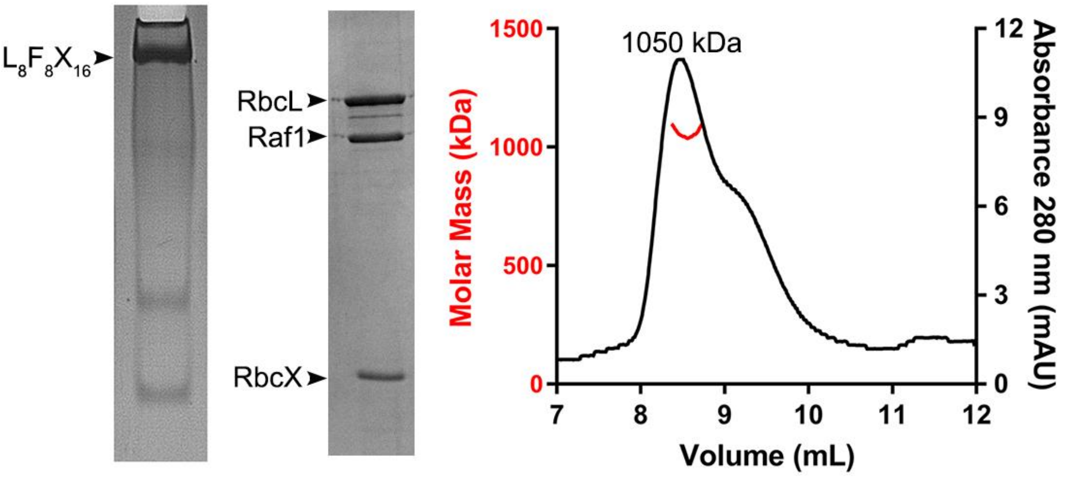

Supplementary Fig. S1 Native-PAGE, SDS-PAGE and Size-exclusion chromatography coupled with multi-angle static light scattering analyses of the ternary complex RbcL-Raf1-RbcX from *Anabaena* sp. PCC 7120. The calculated molecular mass of ~1050 kDa is comparable to that of $L_8F_8X_{16}$.

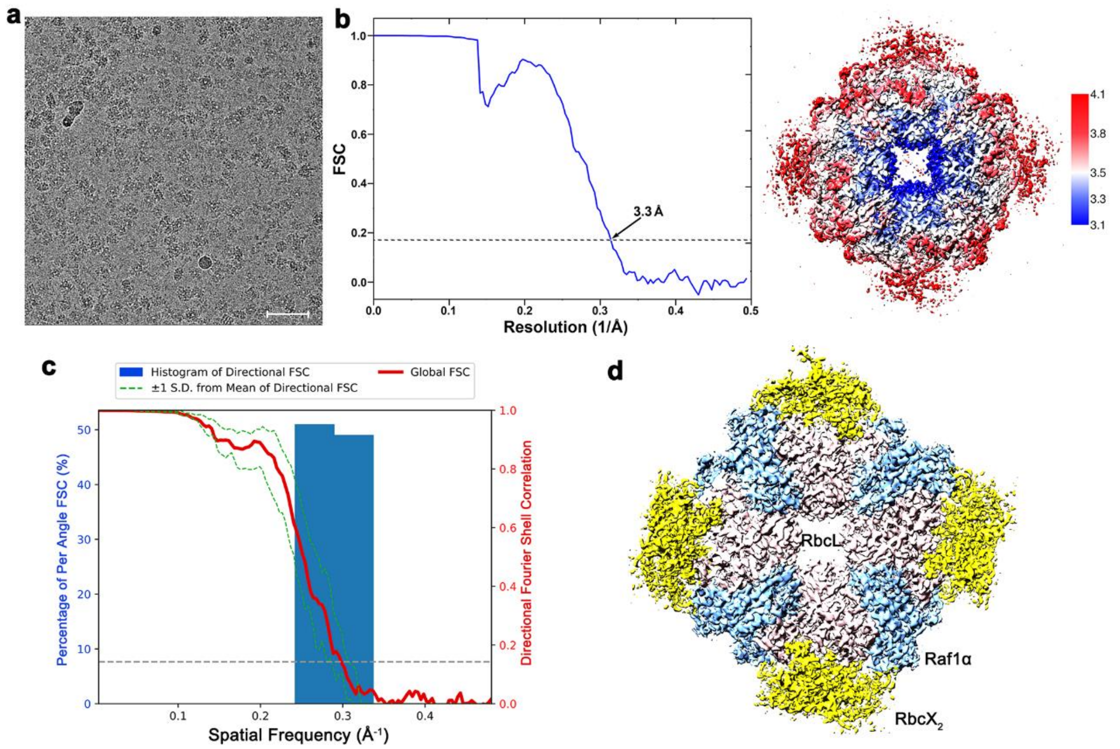

Supplementary Fig. S2 Cryo-EM analyses of $L_8F_8X_{16}$. a A representative cryo-EM image of $L_8F_8X_{16}$ after motion correction and contrast transfer function correction. Scale bar, 50 nm. **b** The gold-standard Fourier shell correlation curve and local resolution map of $L_8F_8X_{16}$. **c** The directional FSC curves and the sphericity value for the map of $L_8F_8X_{16}$. **d** Cryo-EM map of $L_8F_8X_{16}$. The maps corresponding to RbcL, Raf1α and RbcX are colored in pink, marine and yellow, respectively.

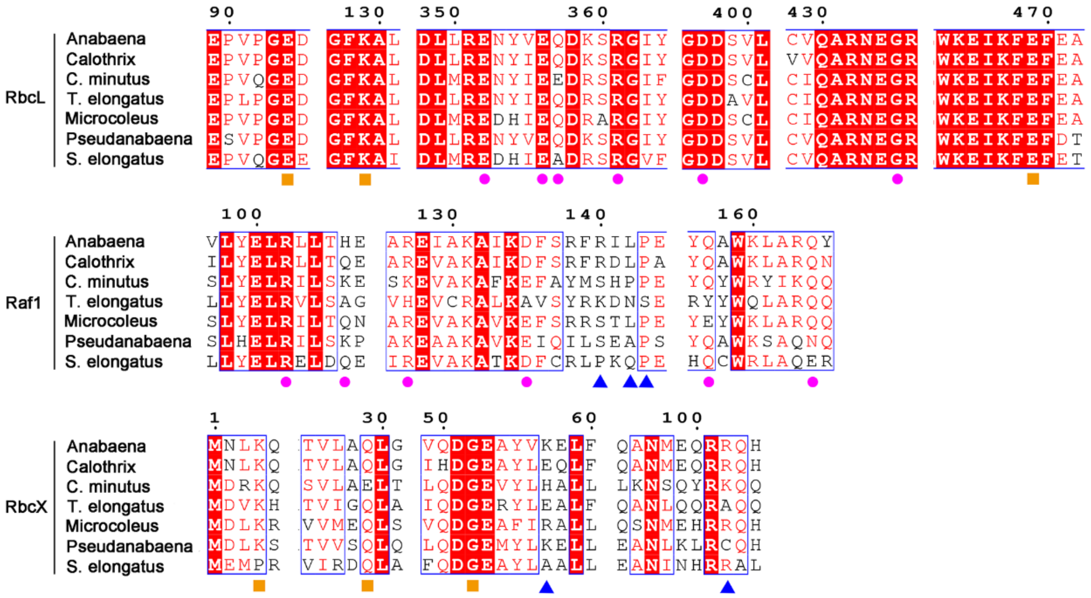

Supplementary Fig. S3 Multiple-sequence alignments of cyanobacterial homologs of RbcL, Raf1 and RbcX, respectively. The interacting residues of RbcL-Raf1α, RbcL-RbcX, and Raf1α-RbcX in the $L_8F_8X_{16}$ structure are marked with magentas circles, orange squares and blue triangles, respectively. The alignments were performed with the programs Clustal Omega and Espript. Anabaena, *Anabaena* sp. PCC 7120; Calothrix, *Calothrix* sp. PCC 7507; C. minutus, *Chamaesiphon minutus* PCC 6605; T. elongatus, *Thermosynechococcus elongatus* BP-1; Microcoleus, *Microcoleus* sp. PCC 7113; Pseudanabaena, *Pseudanabaena* sp. PCC 7367; S. elongatus, *Synechococcus elongatus* PCC 7942.

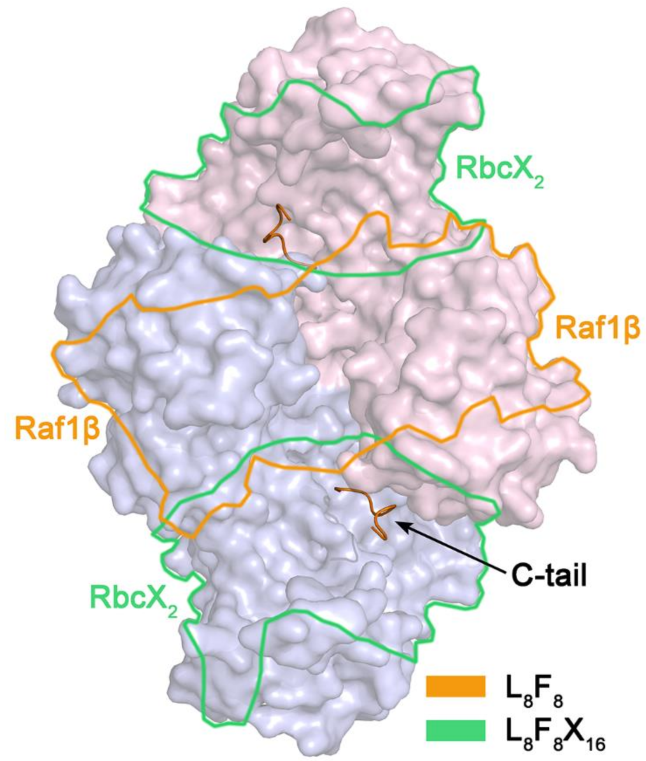

Supplementary Fig. S4 RbcX and Raf1β share partly overlapped binding regions on RbcL. The RbcL dimer is shown as pink and marine semi-transparent surface for each subunit, respectively. The RbcX- and Raf1β-binding regions are indicated by green and orange circles, respectively. The C-tail of Raf1 in the L_8F_8 structure is shown as cartoon and colored in orange.

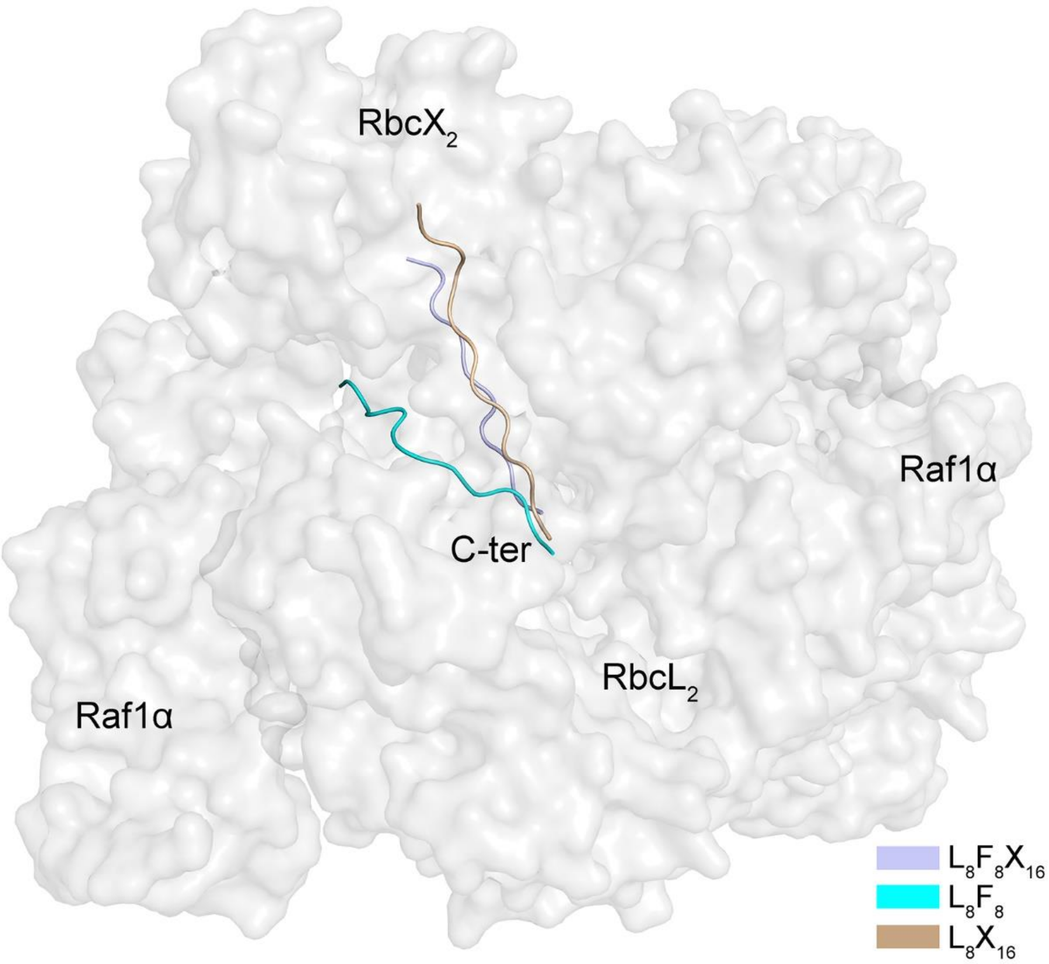

Supplementary Fig. S5 Comparison of RbcL C-terminal peptides (C-ter) in the structures of $L_8F_8X_{16}$, L_8F_8 (PDB, 6KKM) and L_8X_{16} (PDB, 3RG6). The $RbcL_2$, $RbcX_2$ and Raf1α are shown as semi-transparent surfaces and colored in gray. The C-ter of RbcL in three structures are shown as marine, cyan and wheat cartoons, respectively.

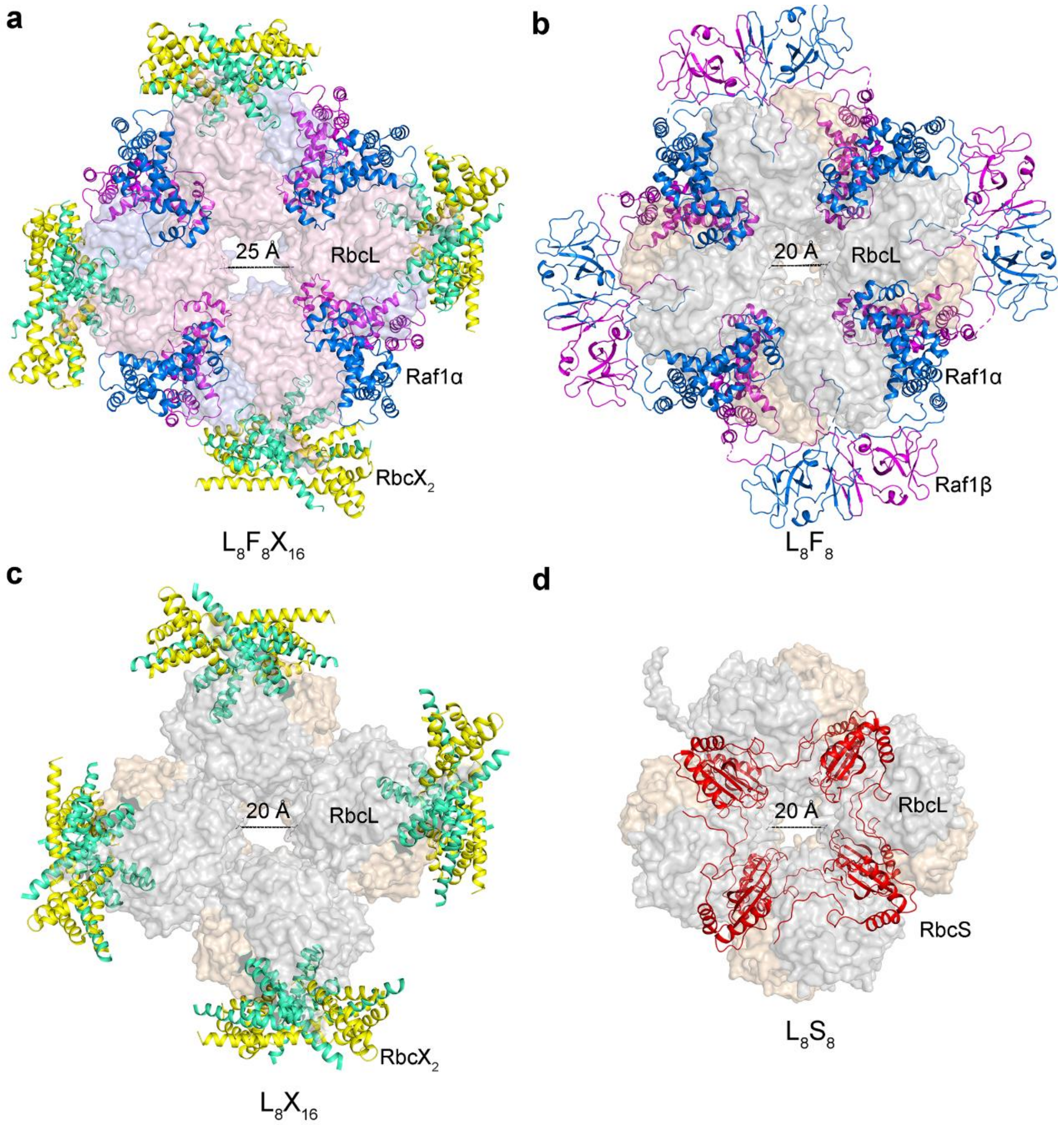

Supplementary Fig. S6 Comparison of $RbcL_8$ central pores in the structures of $L_8F_8X_{16}$, L_8F_8 (PDB, 6KKM), L_8X_{16} (PDB, 3RG6) and L_8S_8 (PDB, 6Z1F). The $RbcL_8$ core is shown as semi-transparent surface, whereas Raf1, RbcX and RbcS are shown as cartoons. The diameter of each central pore is labeled.

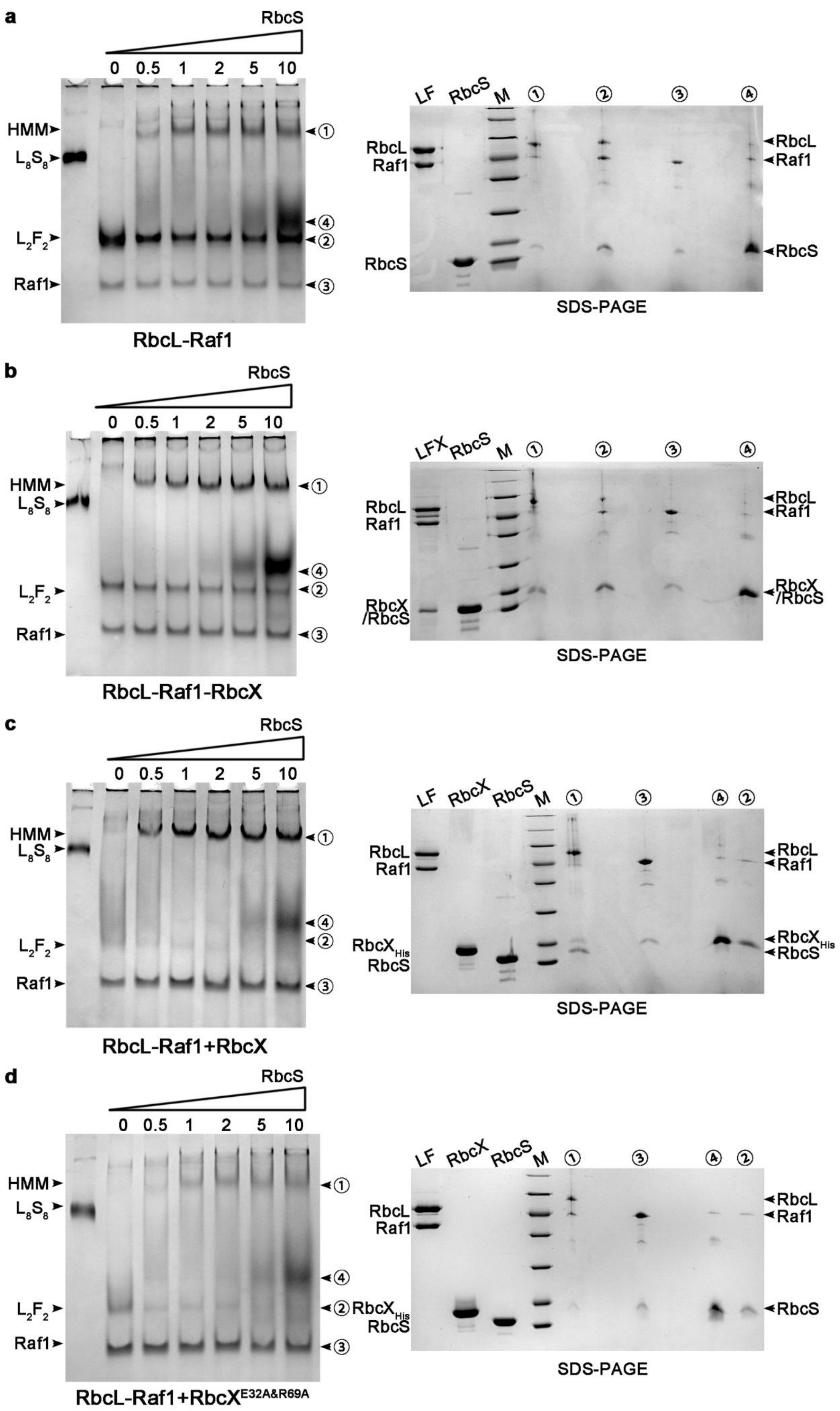
a
RbcS
0 0.5 1 2 5 10
HMM
L_8S_8
L_2F_2
Raf1
①
④
②
③
RbcL-Raf1
LF RbcS M ① ② ③ ④
RbcL
Raf1
RbcS
SDS-PAGE
b
RbcS
0 0.5 1 2 5 10
HMM
L_8S_8
L_2F_2
Raf1
RbcL-Raf1-RbcX
LFX RbcS M ① ② ③ ④
RbcL
Raf1
RbcX
/RbcS
SDS-PAGE
c
RbcS
0 0.5 1 2 5 10
HMM
L_8S_8
L_2F_2
Raf1
RbcL-Raf1+RbcX
LF RbcX RbcS M ① ③ ④ ②
RbcL
Raf1
$RbcX_{His}$
RbcS
SDS-PAGE
d
RbcS
0 0.5 1 2 5 10
HMM
L_8S_8
L_2F_2
Raf1
$RbcL-Raf1+RbcX^{E32A\&R69A}$
LF RbcX RbcS M ① ③ ④ ②
RbcL
Raf1
$RbcX_{His}$
RbcS
SDS-PAGE

Supplementary Fig. S7 Native- and SDS-PAGE analyses of RuBisCO assembly. RbcS proteins at increasing concentrations were added to the solution of RbcL-Raf1 (**a**), RbcL-Raf1-RbcX (**b**), RbcL-Raf1 complexes pre-incubated with 16-fold RbcX (**c**) or $RbcX^{E32A\&R69A}$ (**d**), respectively. The corresponding bands at the last lane of native-PAGE were cut off and further analyzed by SDS-PAGE. The purified *Anabaena* sp. PCC 7120 RbcL-Raf1 (LF), RbcL-Raf1-RbcX (LFX), $RbcX_{His}$ and RbcS proteins were applied as positive controls in SDS-PAGE. $RbcX_{His}$ represents RbcX with a His-tag at the N-terminus, whereas M represents the unstained protein molecular-weight marker (Pierce™).

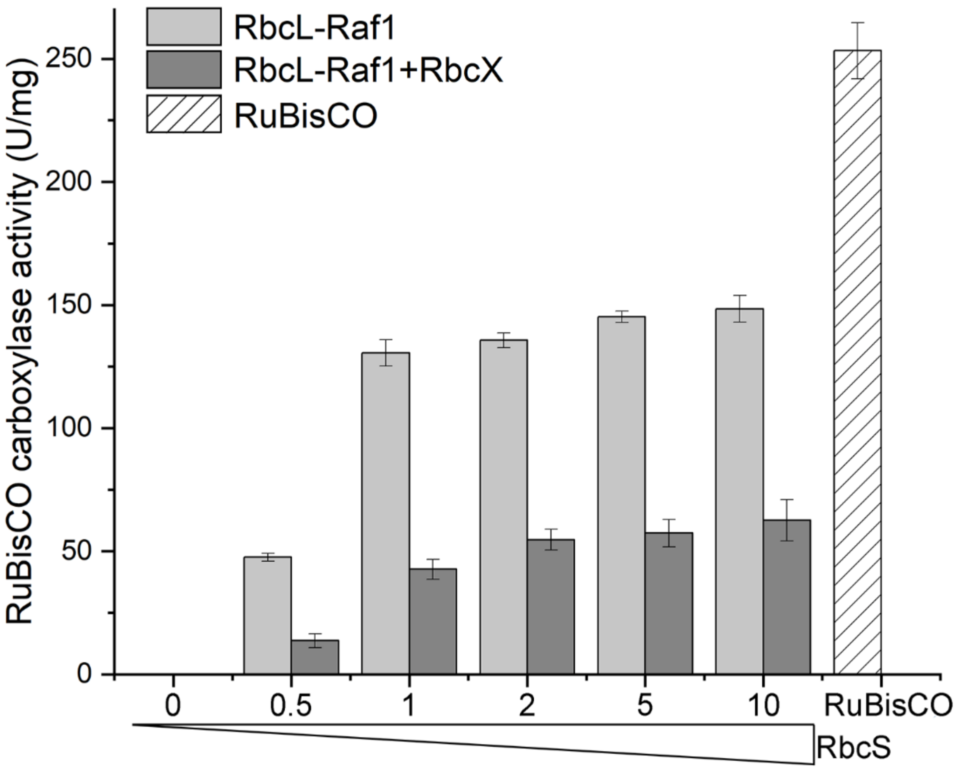

Supplementary Fig. S8 The RuBisCO carboxylase activities of the HMM complexes in Figure 2a and 2c. Three independent assays were performed to calculate the means and standard deviations (SD), and the data are presented as the means ± SD.

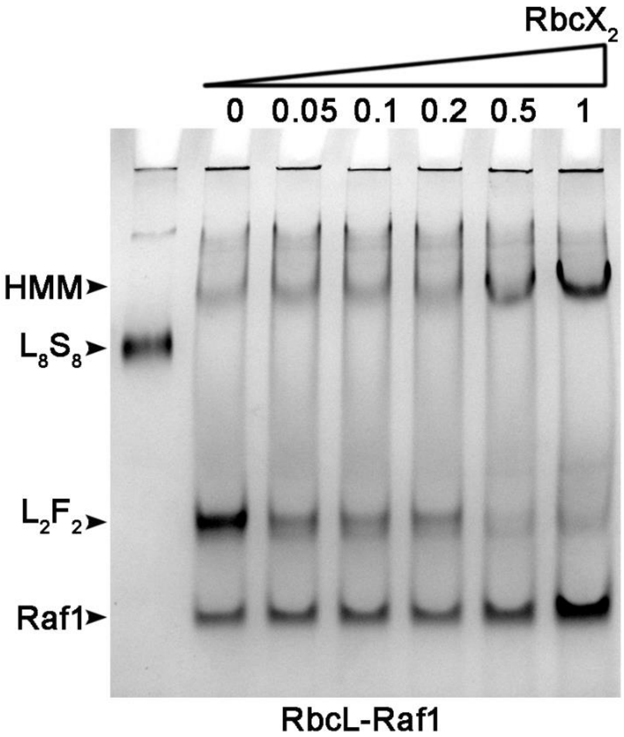

Supplementary Fig. S9 Native-PAGE analysis of RuBisCO assembly. The 0.5 μM solution of RbcL-Raf1 complexes pre-incubated with RbcX proteins at increasing concentrations were added to 4 μM RbcS. The concentrations of $RbcX_2$ are 0, 0.2, 0.4, 0.8, 2 and 4 μM, with the molar ratio of 0, 0.05, 0.1, 0.2, 0.5 and 1-fold $RbcX_2$ (as shown at the top) to RbcL. The assembly intermediates are indicated by arrows on the left of the native-PAGE, in which HMM represents the complexes of high-molecular-mass intermediates. The *Anabaena* sp. PCC 7120 L_8S_8 holoenzyme was used as the positive control in lane 1.

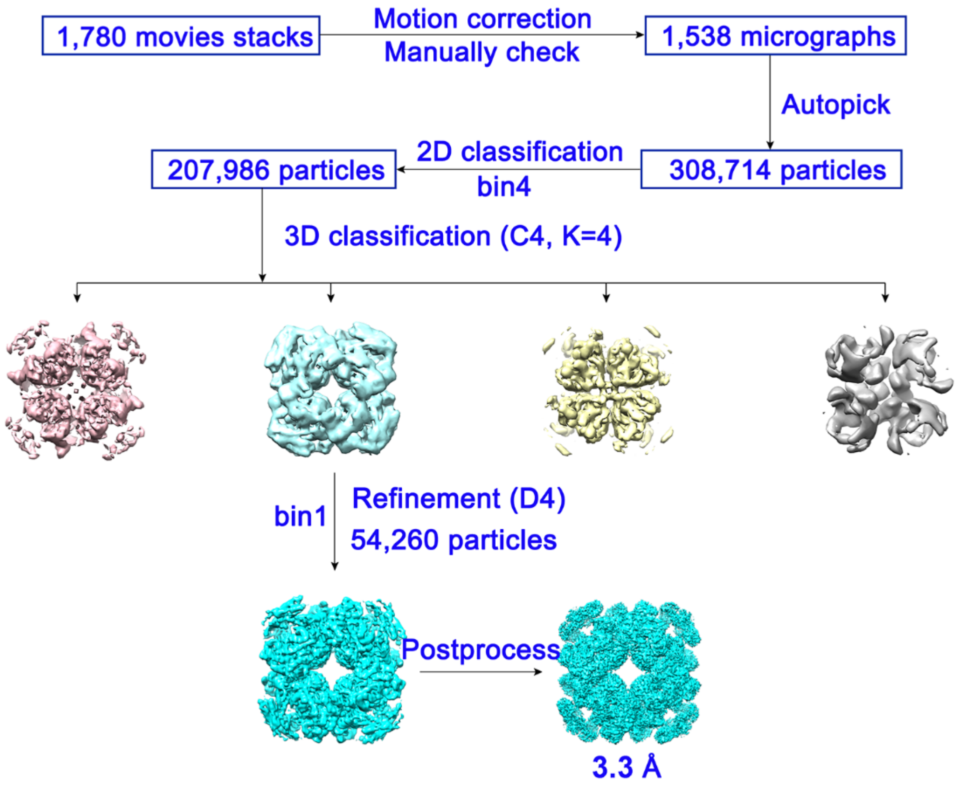

Supplementary Fig. S10 Flowchart for cryo-EM data processing of $L_8F_8X_{16}$. The schematic flowchart illustrates the cryo-EM data processing, classification and reconstruction of $L_8F_8X_{16}$.

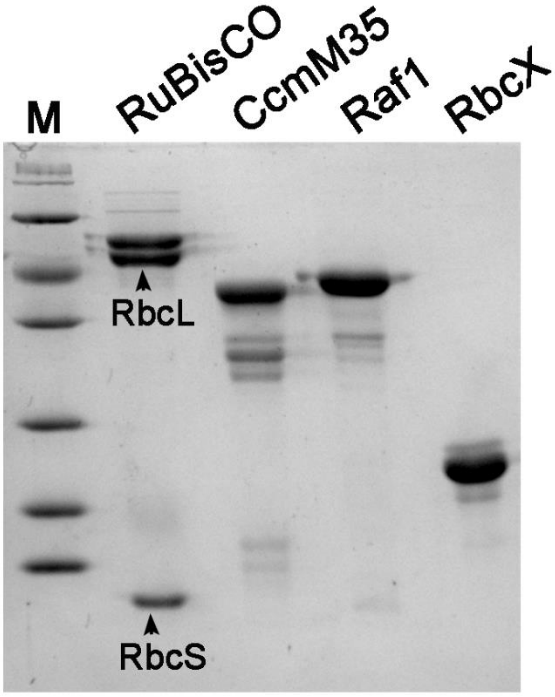

Supplementary Fig. S11 SDS-PAGE analyses of the purified proteins *S. elongatus* PCC 7942 RuBisCO, CcmM35, Raf1 and RbcX that were used in the turbidimetric assays. M represents the unstained protein molecular-weight marker (Pierce™).

Supplementary Table S1. Summary of residues involved in the three interfaces among RbcL, Raf1 and RbcX.

Anabaena sp. PCC 7120 (*Spinach*)		
**RbcL**	**Bonding type**	**Raf1**
E352 (D351)	Hydrogen bond/Salt bridge	R127 (R211)
E356 (E355)	Hydrogen bond	Q157 (T244)
Q357 (K356)	Hydrogen bond	Q164 (E251)
R361 (R360)	Hydrogen bond/Salt bridge	D135 (E219)
D397 (D396)	Hydrogen bond/Salt bridge	R102 (R186)
G435 (G434)	Hydrogen bond	H106 (T190)
**RbcL**	**Bonding type**	**RbcX**
E94 (E93)	Salt bridge	K4 (E102)
K129′ (K128)	Hydrogen bond	Q29 (Q127)
E471 (P470)	Hydrogen bond	G52 (G151)
**RbcX**	**Bonding type**	**Raf1**
K57 (R156)	Hydrogen bond	R140 (Q224)
R102 (R202)	Hydrogen bond	L142 (E226)
R102 (R202)	Hydrogen bond	P143 (P227)

Supplementary Table S2. The cyanobacterial strains, plasmids and protein sequences used in this study.

	Recombinant plasmids	Proteins	Tag sequences
***Anabaena* sp. PCC 7120**	pET19-His-RbcL	His-RbcL	MGHHHHHHHHHHSSG-RbcL
	pCDFduet-GroEL-GroES-RbcX-Raf1	GroEL	without tag
		GroES	without tag
		Raf1	without tag
		RbcX	without tag
	pET19-His-RbcL-Raf1	His-RbcL	MGHHHHHHHHHHSSG-RbcL
		Raf1	without tag
	pET19-His-RbcL-Raf1$^{\Delta C8}$	His-RbcL	MGHHHHHHHHHHSSG-RbcL
		Raf1$^{\Delta C8}$	without tag
	pCDFduet-GroEL-GroES	GroEL	without tag
		GroES	without tag
	pET19-His-RbcX	His-RbcX	MGHHHHHHHHHHSSG-RbcX
	pET19-His-RbcX$^{E32A\&R69A}$	His-RbcX$^{E32A\&R69A}$	MGHHHHHHHHHHSSG-RbcX$^{E32A\&R69A}$
	pET19-His-RbcS	His-RbcS	MGHHHHHHHHHHSSG-RbcS
	pET-Flag-RbcS	Flag-RbcS	MGDYKDDDDKH-RbcS
	pET19-His-CcmM35	His-CcmM35	MGHHHHHHHHHHSSGHIDDDDKH-CcmM35
***S. elongatus* PCC 7942**	pET19-His-RbcL-Raf1	His-RbcL	MGHHHHHHHHHHSSG-RbcL
		Raf1	without tag
	pCDFduet-GroEL-GroES-RbcS	GroEL	without tag
		GroES	without tag
		RbcS	without tag
	pET19-His-Raf1	His-Raf1	MGHHHHHHHHHHSSG-Raf1
	pET19-His-RbcX	His-RbcX	MGHHHHHHHHHHSSG-RbcX
	pET19-His-CcmM35	His-CcmM35	MGHHHHHHHHHHSSGHIDDDDKH-CcmM35
**Chimeric plasmid**	pCDFduet-GroEL-GroES-RbcX-Raf1	GroEL	without tag (*Anabaena* sp. PCC 7120)
		GroES	without tag (*Anabaena* sp. PCC 7120)
		Raf1	without tag (*S. elongatus* PCC 7942)
		RbcX	without tag (*Anabaena* sp. PCC 7120)

Protein sequences (UniProt ID)

***Anabaena* sp. PCC 7120**

RbcL (P00879)

MSYAQTKTQTKSGYKAGVQDYRLTYYTPDYTPKDTDILAAFRVTPQPGVPFEEAAAAV
AAESSTGTWTTVWTDLLTDLDRYKGRCYDIEPVPGEDNQFIAYIAYPLDLFEEGSITNVL

TSIVGNVFGFKALRALRLEDIRFPVAYIKTFQGPPHGIQVERDKLNKYGRPLLGCTIKPKL
GLSAKNYGRAVYECLRGGLDFTKDDENINSAPFQRWRDRFLFVADAITKAQAETGEIK
GHYLNVTAPTCEEMLKRAEYAKELKQPIIMHDYLTAGFTANTTLARWCRDNGVLLHIH
RAMHAVIDRQKNHGIHFRVLAKALRLSGGDHIHTGTVVGKLEGERGITMGFVDLLREN
YVEQDKSRGIYFTQDWASLPGVMAVASGGIHVWHMPALVEIFGDDSVLQFGGGTLGH
PWGNAPGATANRVALEACVQARNEGRNLAREGNDVIREAAKWSPELAVACELWKEIK
FEFEAMDTV

Raf1 (Q8YLP6)

MTELPPNAPNPENATNELAQELLRKLRQKQGNWVEWGQAIASLQKSGYNPQDIFEATG
FEPVQQNQVIVGSQVYNSLEKSGASAATLAHYATRGSDVLYELRLLTHEERAAAGDLT
FTHKVDADEAREIAKAIKDFSRFRILPEGFSNHPGDAVAYQAWKLARQYSDLQERSRLI
ARGLRFAHSETARKQIEQLLVDFTVVSQRPAPIPPFFRFDTEDELPRIVPVVGQLPLKAEE
LKAVPLVEEIEPFRLVKFSGEQAWVALPGWQVLLAAEDPVTILATSDRFPKQNQTEPGP
VLVVVDRSQREWNDFSYFVVDHDGELDFQWFETKPEFPILGKVIILVRPRRILDENVTK
DSWQIDE

RbcX (O86418)

MNLKQIAKDTAKTLQSYLTYQALMTVLAQLGETNPPLALWLHTFSVGKVQDGEAYVK
ELFREQPDLALRIMTVREHIAEEVAEFLPEMVRSGIQQANMEQRRQHLERMTHLSLSNP
SPESEQQTISDTDWDH

RbcS (P06514)

MQTLPKERRYETLSYLPPLTDVQIEKQVQYILSQGYIPAVEFNEVSEPTELYWTLWKLPL
FGAKTSREVLAEVQSCRSQYPGHYIRVVGFDNIKQCQILSFIVHKPSRY

GroEL (Q8YQZ8)

MAKRIIYNENARRALERGIDILAEAVAVTLGPKGRNVVLEKKFGAPQIVNDGVTIAKEIE
LEDHIENTGVALIRQAASKTNDAAGDGTTTATVLAHAIVKEGLRNVAAGANAILLKRGI
DKATGFLVDRIKEHARPVEDSKSIAQVGSISAGNDDEVGQMIAEAMDKVGKEGVISLEE
GKSVTTELEITEGMRFDKGYISPYFATDPERMEAIFDEPFLLLTDKKIALVQDLVPVLEQ
VARAGRPLVIIAEDIEKEALATLVVNRLRGVLNVAAVKAPGFGDRRKAMLEDIAILTGG
QLITEDAGLKLENTKLESLGKARRITITKDSTTIVAEGNDVAVKGRVEQIRRQMEETESS
YDKEKLQERLAKLSGGVAVVKVGAATETEMKDKKLRLEDAINATKAAVEEGIVPGGG
TTLAHLTPELEVWANSNLKDEELTGALIVARALPAPLKRIAENAGQNGAVIAERVKEKA
FNVGFNAATNEFVDMFEAGIVDPAKVTRSALQNAASIAGMVLTTECIVVDKPEPKDNA
PAGAGAGGGDFDY

GroES (Q8YQZ9)

MAAVSLSVSTVKPLGDRVFVKVSASEEKTAGGLYLPDTAKEKPQVGEVVALGAGKRN
DDGSRQELEVKVGDKVLYSKYAGTDVKLGTEEYVLLSEKDILAVVG

CcmM58 (Q8YYI3) (CcmM35: Val224–Pro555)

MAVRSTAAPPTPWSRSLAEAQIHESAFVHPFSNIIGDVHIGANVIIAPGTSIRADEGTPFHIG

ENTNIQDGVVIHGLEQGRVVGDDNKEYSVWVGSSASLTHMALIHGPAYVGDNSFIGFRST
VFNAKVGAGCIVMMHALIKDVEVPPGKYVPSGAIITNQKQADRLPDVQPQDRDFAHHVI
GINQALRAGYLCAADSKCIAPLRNDQVKSYTSTTVIGLERSSEVASNSLGAETIEQVR YLL
EQGYKIGSEHVDQRRFRTGSWTSCQPIEARSVGDALAALEACLADHSGEYVRLFGIDPKG
KRRVLETIIQRPDGVVAGSTSFKAPASNTNGNGSYHSNGNGNGYSNGATSGKVSAETVDQ
IRQLLAGGYKIGTEHVDERRFRTGSWNSCKPIEATSAGEVVAALEECIDSHQGEYIRLIGID
PKAKRRVLESIIQRPNGQVAPSSSPRTVVSASSASSGTATATATRLSTEVVDQVRQILGGGY
KLSIEHVDQRRFRTGSWSSTGAISATSEREAIAVIEASLSEFAGEYVRLIGID PKAKRRVLET
IIQRP

***S. elongatus* PCC 7942**

RbcL (Q31NB3)

MPKTQSAAGYKAGVKDYKLTYYTPDYTPKDTDLLAAFRFSPQPGVPADEAGAAIAAES
STGTWTTVWTDLLTDMDRYKGKCYHIEPVQGEENSYFAFIAYPLDLFEEGSVTNILTSIV
GNVFGFKAIRSLRLEDIRFPVALVKTFQGPPHGIQVERDLLNKYGRPMLGCTIKPKLGLS
AKNYGRAVYECLRGGLDFTKDDENINSQPFQRWRDRFLFVADAIHKSQAETGEIKGHY
LNVTAPTCEEMMKRAEFAKELGMPIIMHDFLTAGFTANTTLAKWCRDNGVLLHIHRA
MHAVIDRQRNHGIHFRVLAKCLRLSGGDHLHSGTVVGKLEGDKASTLGFVDLMREDHI
EADRSRGVFFTQDWASMPGVLPVASGGIHVWHMPALVEIFGDDSVLQFGGGTLGHPW
GNAPGATANRVALEACVQARNEGRDLYREGGDILREAGKWSPELAAALDLWKEIKFEF
ETMDKL

RbcS (Q31NB2)

MKTLPKERRFETFSYLPPLSDRQIAAQIEYMIEQGFHPLIEFNEHSNPEEFYWTMWKLPLF
DCKSPQQVLDEVRECRSEYGDCYIRVAGFDNIKQCQTVSFIVHRPGRY

Raf1 (Q31Q05)

MREFTPTTLSEEERQELLGQLRRKEGRWLAWARACQTLLKNGLNPQTLFEATGFEPIQQ
NQITVAMQVYDSILRQDPPAHVRETYQEWGSDLLYELRELDQEQRSLCAQLALERKLD
ADQIREVAKATKDFCRLPKQPENFDRHPGDAVAHQCWRLAQERTDLTERSRLIARGLQ
FAQSAGARALIEALLLDLSGVPSRKPPMLPIYRLETEEDLPRLLPFAGTLPLSSSQIEAIAA
VEAEGPFGLVSSPQGQQWLALPGWQAILTAEDPIACLEQIDRLPNAPEGPTEAVVLVVD
RADRDWDADHFFLVEQAEGARIQWSPSAIAAPILGRLVLILRPKRVLDEAAIATPWQFE
E

RbcX (Q31N04)

MASTQRAKPMEMPRISRDTARMLVNYLTYQAVCVIRDQLAETNPAGAYRLQVFSAEFSFQ
DGEAYLAALLNHDRELGLRVMTVREHLAEHILDYLPEMTI AQIQEANINH RRALLERLTG
LGAEPSLPET EVSDRPSDSA TPDDASNASHAD

GroEL (P22879)

MAKRIIYNENARRALEKGIDILAEAVAVTLGPKGRNVVLEKKFGAPQIINDGVTIAKEIE
LEDHIENTGVALIRQAASKTNDAAGDGTTTATVLAHAVVKEGLRNVAAGANAILLKRG
IDKATNFLVEQIKSHARPVEDSKSIAQVGAISAGNDFEVGQMIADAMDKVGKEGVISLE

EGKSMTTELEVTEGMRFDKGYISPYFATDTERMEAVFDEPFILITDKKIGLVQDLVPVLEQVARAGRPLVIIAEDIEKEALATLVVNRLRGVLNVAAVKAPGFGDRRKAMLEDIAVLTGGQLITEDAGLKLDTTKLDQLGKARRITITKDNTTIVAEGNEAAVKARVDQIRRQIEETESSYDKEKLQERLAKLSGGVAVVKVGAATETEMKDRKLRLEDAINATKAAVEEGIVPGGGTTLAHLAPQLEEWATANLSGEELTGAQIVARALTAPLKRIAENAGLNGAVISERVKELPFDEGYDASNNQFVNMFTAGIVDPAKVTRSALQNAASIAAMVLTTECIVVDKPEPKEKAPAGAGGGMGDFDY

GroES (P22880)

MAAVSLSVSTVTPLGDRVFVKVAEAEEKTAGGIILPDNAKEKPQVGEIVAVGPGKRNDDGSRQAPEVKIGDKVLYSKYAGTDIKLGNDDYVLLSEKDILAVVA

CcmM58 (Q03513) (CcmM35: Val216 – Pro539)

MPSPTTVPVATAGRLAEPYIDPAAQVHAIASIIGDVRIAAGVRVAAGVSIRADEGAPFQVGKESILQEGAVIHGLEYGRVLGDDQADYSVWIGQRVAITHKALIHGPAYLGDDCFVGFRSTVFNARVGAGSVIMMHALVQDVEIPPGRYVPSGAIITTQQQADRLPEVRPEDREFARHIIGSPPVIVRSTPAATADFHSTPTPSPLRPSSSEATTVSAYNGQGRLSSEVITQVRSLLNQGYRIGTEHADKRRFRTSSWQPCAPIQSTNERQVLSELENCLSEHEGEYVRLLGIDTNTRSRVFEALIQRPDGSVPESLGSQPVAVASGGGRQSSYASVSGNLSAEVVNKVRNLLAQGYRIGTEHADKRRFRTSSWQSCAPIQSSNERQVLAELENCLSEHEGEYVRLLGIDTASRSRVFEALIQDPQGPVGSAKAAAAPVSSATPSSHSYTSNGSSSSDVAGQVRGLLAQGYRISAEVADKRRFQTSSWQSLPALSGQSEATVLPALESILQEHKGKYVRLIGIDPAARRRVAELLIQKP

Supplementary Table S3. Cryo-EM parameters, data collection and refinement statistics.

	$L_8F_8X_{16}$ (EMD-33524) (PDB 7XSD)
Data collection and processing	
Magnification	22,500
Voltage (keV)	300
Electron exposure (e^-/Å^2)	50
Defocus range (μm)	-1.0 ~ -2.0
Pixel size (Å)	1.01
Symmetry imposed	D4
Initial particle images (no.)	308,714
Final particle images (no.)	54,260
Map resolution (Å)	3.3
FSC threshold	0.143
Map resolution range (Å)	2.02~999
Refinement	
Real-space correlation coefficient	0.70
Initial model used (PDB code)	6KKM, 2PEO
Model resolution (Å)	3.3
Map sharpening B factor (Å^2)	-115.2
Model composition	
Non-hydrogen atoms	49,848
Protein residues	6,272
Waters	0
RMS deviation from ideality	
Bond lengths (Å)	0.009
Bond angles (°)	1.257
Validation	
MolProbity score	1.83
Clash score	6.72
Poor rotamers (%)	0.77
Ramachandran statistics	
Favored regions (%)	92.7
Allowed regions (%)	7.2
Outliers (%)	0.1
